# Human Vital Signs Detection Methods and Potential Using Radars: A Review

**DOI:** 10.3390/s20051454

**Published:** 2020-03-06

**Authors:** Mamady Kebe, Rida Gadhafi, Baker Mohammad, Mihai Sanduleanu, Hani Saleh, Mahmoud Al-Qutayri

**Affiliations:** 1 System on Chip Center, Khalifa University, P.O. Box 127788, Abu Dhabi, UAE; mamady.kebe@ku.ac.ae (M.K.); rgadhafi@ud.ac.ae(R.G.); mihai.sanduleanu@ku.ac.ae (M.S.); hani.saleh@ku.ac.ae (H.S.); mahmoud.alqutayri@ku.ac.ae (M.A.-Q.); 2 College of Engineering & IT (CEIT), University of Dubai, P.O. Box 14143, Dubai, UAE

**Keywords:** heart rate, radars, respiration rate, vital signs, sensors

## Abstract

Continuous monitoring of vital signs, such as respiration and heartbeat, plays a crucial role in early detection and even prediction of conditions that may affect the wellbeing of the patient. Sensing vital signs can be categorized into: contact-based techniques and contactless based techniques. Conventional clinical methods of detecting these vital signs require the use of contact sensors, which may not be practical for long duration monitoring and less convenient for repeatable measurements. On the other hand, wireless vital signs detection using radars has the distinct advantage of not requiring the attachment of electrodes to the subject’s body and hence not constraining the movement of the person and eliminating the possibility of skin irritation. In addition, it removes the need for wires and limitation of access to patients, especially for children and the elderly. This paper presents a thorough review on the traditional methods of monitoring cardio-pulmonary rates as well as the potential of replacing these systems with radar-based techniques. The paper also highlights the challenges that radar-based vital signs monitoring methods need to overcome to gain acceptance in the healthcare field. A proof-of-concept of a radar-based vital sign detection system is presented together with promising measurement results.

## 1. Introduction

The four major vital signs are body temperature (BT), heart rate (HR), breath rate (BR) and blood pressure (BP). They provide almost a complete picture of individuals’ body vital functions and help to assess their general physical health. The assessment of body temperature is relatively simple, low cost and does not often require continuous monitoring. On the other hand, detection and monitoring of breath rate and heart rate usually require complex systems involving sensors and computers that are physically connected. Breath rate or breath frequency determines the number of respiration cycles performed by an individual in one minute while the heart rate or pulse rate corresponds to the number of heart beats per minute. The standard breath rate and pulse rate of a healthy adult individual ranges between 12–20 breaths per minute and 60–100 beats per minute, respectively [1,2]. For children these ranges change to 17–40 breaths per minute and 70–190 beats per minute for BR and HR, respectively. Any abnormality to the standard cardio-pulmonary rates may indicate a sign of physical or mental stress. There are two main techniques of the cardio-pulmonary vital signs detection: the contact-based methods and the contactless-based methods using radars.

Monitoring of human cardio-pulmonary rates exhibits a variety of applications from medical diagnostics to fitness assessment and emotion recognition. Heart and respiration rates monitoring can be used to predict certain pulmonary and cardiovascular diseases, which account for more than 31% of all deaths worldwide [3]. The obstructive sleep apnea syndrome (OSAS) and sudden infant death syndrome (SIDS) are two major conditions causing high mortality in both infants and adults. SIDS is the leading cause of death of infants under 1 year old in the developed world [4]. In 2016, SIDS accounted for 38% of all sudden unexpected infant death (SUID) in the United States [4]. Furthermore, a new study conducted in 2018 estimated that nearly a billion people are affected by OSAS around the world [5], corresponding to 10 times the number previously reported by the World Health Organization (WHO) in 2007. Therefore, continuous monitoring of infants, elderly or injured patients is necessary, especially for homecare applications. Monitoring of vital signs is essential in the early detection of both OSAS [6] and SIDS [7]. Furthermore, it has been proven that both the pulse rate and respiratory rate are fundamental predictors of cardiac arrest [8]. A study in [8] shows that the difference in BR and HR in individuals at risk of cardio-pulmonary arrest and healthy individuals is more significant than the difference in other vital signs such as BP and BT. Therefore, it is crucial to monitor HR and BR for diagnosis of this fatal condition. Moreover, vital signs monitoring can be used to monitor athletes and non-athletes during exercise. Since cardiac health issues can be complex and do not exhibit any apparent symptoms [9], it is vital to monitor the cardio-respiratory rates in order to adjust the appropriate intensity of exercise for the body. In addition, exercise-induced dyspnea and chronic obstructive pulmonary disease (COPD) are proven to have strong correlation with BR [10,11]. Finally, vital signs monitoring can be applied in the assessment of physical and psychological stress of individuals. In [12], the physical conditions of firefighters during work have been examined and compared to the normal physiological conditions using their vital signs data. Mood disorder and stress are shown to be related to the respiration rate, heart rate and heart rate variability (HRV) [13]. BR was demonstrated to be related to cognitive load after vital signs measurement experiments were carried on high-demanding task performers such as pilots, soldiers and surgeons.

In this paper, we review different methods used in contact-based (Section 2) and contactless radar-based (Section 3) cardio-pulmonary signals’ detection and monitoring as well as their challenges in biomedical applications. Furthermore, state-of-the-art solutions for challenges in vital signs detection using radars are provided in Section 3 as they are promising technologies to potentially replace traditional contact-based vital signs sensing. In Section 4, experimental results of a continuous-wave radar is presented to demonstrate the feasibility of wireless sensing of human vital signs. Section 5 summarizes the review of the two vital signs detection methods as well as future works on the radar-based vital signs acquisition.

## 2. Conventional Contact-Based Vital Signs Acquisition

This section reviews some of the major contact-based methods for detecting human respiratory and cardiac rates. The review highlights the fundamental principles of the various methods as well as some of their shortcomings. The contact-based methods addressed in this section include electrocardiography, photoplethysmography, airflow sensing, chest-wall mechanical displacement sensing, blood pressure sensing and sound-based sensing. We will explain each type in the next sections

### 2.1. Electrocardiography

Electrocardiography is a process of acquiring the electrical activity of the heart resulting from the action potential generated by heartbeats. It is based on measuring the potential difference between at least two points on the body surface of the subject [14]. The signal obtained from an electrocardiography is called the electrocardiogram (ECG). ECG monitoring systems are crucial for the diagnosis of heart conditions such as myocardial ischemia and arrhythmia [15]. Even though ECG systems are mainly used to monitor heart signals, breathing rate can be estimated from the ECG data [16,17]. Called ECG-derived respiration (EDR), this technique is based on a process named sinus arrhythmia [18]. There are two common ECG systems, namely the 12-lead clinical ECG system and the portable ambulatory ECG system.

**Figure 1:**
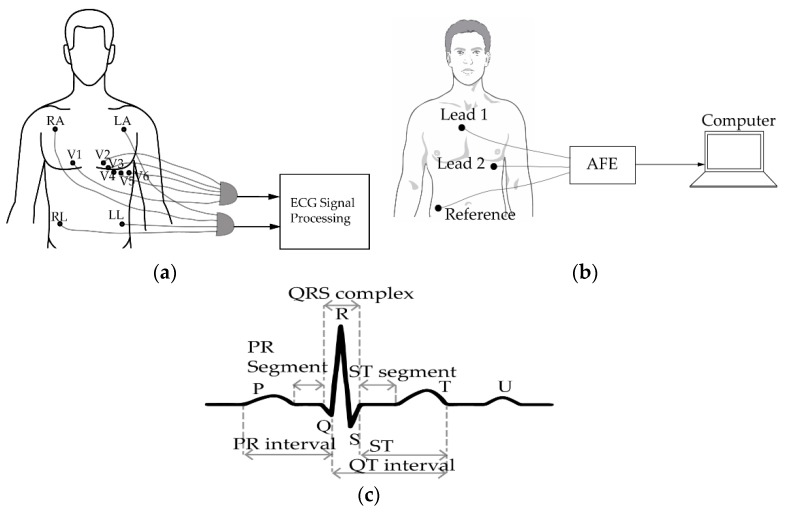
(**a**) Twelve-lead clinical electrocardiogram (ECG) system; (**b**) ambulatory ECG system; (**c**) representation of ECG signal.

In the 12-lead ECG system, 10 electrodes are attached to the surface of limbs and chest of the subject, generating 12 groups of signals [19] (Figure 1a). Electrodes denominated by RA (right arm) and LA (left arm) are usually attached either to the wrist or the upper chest area of the patient. On the other hand, the right leg (RL) and left leg (LL) electrodes are attached to the leg or the lower peripheral stomach areas. Data obtained from the leads are processed by a computer and displayed on a screen. Conventional clinical ECG are directly connected to the signal-processing unit using cables. However, new-generation clinical ECGs have Bluetooth or ZigBee facilities, eliminating the need for cabling [20]. However, the need to place electrodes on the skin of the patient persists. Even though the 12-lead ECG system provide more accurate cardiac signal than the portable ambulatory ECG system, its bulky nature and use of many electrodes and sensors impede its usage outside clinical applications. Despite its relatively high cost, the traditional clinical ECG require a trained person to operate.

On the other hand, ambulatory ECG systems have typically three leads or fewer and are smaller in size, as shown in Figure 1b. The bio-potential signals sensed by the electrodes pass through an analog front end (AFE) unit, where it is subjected to filtering and amplification before being processed by a digital signal processing (DSP) unit. This type of ECG system can be used in domestic settings, unlike the conventional clinical ECG systems [21]. Ambulatory ECG systems can be designed to have low power and size [22,23], although they are less accurate compared to the clinical ECG systems.

A typical ECG waveform is represented in Figure 1c. P wave designates the sequential activation of the right and left atria, while T and U waves represent ventricular repolarization and the repolarization of the interventricular septum, respectively. The QRS complex (combination of ‘Q’ wave, ‘R’ wave and ‘S’ wave) represents the simultaneous activation of the right and left ventricles [24].

Traditional ECG systems use hydrogel between the contact electrodes and the skin [21] to increase the sensitivity of the system. Nevertheless, because of its toxic nature, the conductive gels cause skin irritation of patients [25]. Furthermore, some patients may be allergic to the acrylic adhesive, which is present in the popular disposable conductive hydrogel-based ECG electrodes [26,27]. Therefore, the wet electrodes method is not suitable for long-term and frequent monitoring of patients. Many recently developed ECG systems use dry capacitive electrodes [18,21,28,29]. Since the dry-electrode sensor does not require any interfacial material, it is more suitable for long-term monitoring than the wet-electrode counterpart. However, the dry electrodes have high electrode tissue impedance (ETI) due to poor contact with the skin and are prone to motion artifacts [20]. Moreover, standard placement of the electrodes must be followed in order to obtain accurate signals [30,31]. Other factors that affect the accuracy of the ECG signal include the motion artifact (MA) of the lead electrodes and change in impedance of the electrodes due to the respiration, which causes an amplitude modulation of the ECG data [32,33]. The inhaling and exhaling process during respiration cause an increase and decrease of the heart rate, respectively and, hence, produce a frequency modulation in the ECG signal [34] for the detection of heartbeat. Clinical ECG systems remain the gold standard method of measuring the heart rate and heart rate variability. Nevertheless, they are bulky and expensive. A relatively cheaper way of acquiring vital signs involve photoplethysmography, which is discussed next.

### 2.2. Photoplethysmography

**Figure 2:**
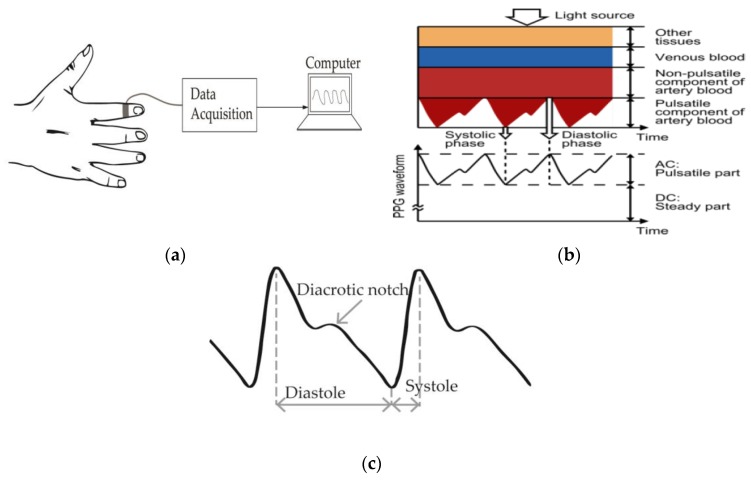
(**a**) Photoplethysmogram (PPG) measurement setup; (**b**) effect of light on different tissues of human body [37]; (**c**) description of PPG waveform.

Photoplethysmography is a technique that uses optical means to instantaneously measure the changes in volume in human tissue [35]. The time-varying signal obtained from photoplethysmography is referred to as the photoplethysmogram (PPG). At least one light-emitting-diode (LED) in direct contact with the tissue is used to emit light with wavelengths between 500 nm and 600 nm, corresponding to the green and yellow regions [36,37]. The green light is widely used for HR acquisition. However, many photoplethysmography setups use the red and infra-red optical regions for blood oxygen monitoring [38,39,40]. A portion of the emitted light is absorbed by human tissue while the other portion is reflected. A photo-detector (PD) is used to record the intensity of the received light. This intensity changes during the systolic and diastolic phases of the cardiac cycle [37]. The PD can be placed either at the same side of the light emitter or the opposite side, depending on whether the acquisition is in reflectance-mode or transmission-mode [41]. Since the attenuation from the body tissue is high at the low optical wavelength region, PPG systems employing the green-yellow regions are better used in reflectance-mode operations, whereas transmission-mode operation is preferred for red and infrared optical signals. However, these two methods differ less in terms of the measurement accuracy of heart rate or heart rate variability, even though higher accuracy blood oxygen measurement is obtained in transmission mode. Pulse oximetry uses the transmission-mode because the venous oscillations are less accentuated in this mode [42] and; hence a higher signal-to-noise ratio (SNR) signal is produced. As represented in Figure 2a, the oximeter probe sensor can be attached to the finger of the subject or any other peripheral sites including ears and toes. Since the wavelength of the light is proportional to the penetration depth, infra-red (IR) light can be used for deep-tissue blood flow measurement. An AFE is used to improve the signal quality before sending to computer wirelessly or via cabling for signal processing. The change in blood volume becomes more significant in the arteries, as shown in Figure 2b.

Moreover, the respiration rate can be estimated from the PPG data since breathing causes an amplitude and frequency modulation of the received signal [34,41], even though the PPG signal is mainly used to estimate the heart rate and blood oxygen saturation. Furthermore, a baseline wander or direct-current (DC) offset is caused by the respiratory cycles. The reflectance mode is often used in the estimation of breath rate since it is more sensitive to venous pressure during respiration [41]. A typical waveform of the PPG is shown in Figure 2c. The modulation due to breathing can be clearly seen as well as the pulsatile components of the heartbeat.

Despite being cost-effective, PPG systems most often produce signals that are affected by several factors including the measuring site, ambient temperature and the posture of the subject [35,37,43]. In addition, the accuracy of the PPG raw signal can be undermined by the MA of the subject [43].

Many recent works on PPG remote monitoring are based on the use of a camera, which can be from a cellphone or a laptop [24,44,45,46]. Called video plethysmography (VPG), this technique uses ambient light as the emitter source and the camera as the photodetector [32]. The ambient optical illumination can be natural light or compact fluorescent [44]. The red-green-blue (RGB) components of the camera are separated and processed using different signal-processing methods [44]. Even though the VPG offers the ability to monitor the heart rate remotely, it can only provide rough value of the heart rate rather than beat-to-beat plethysmography using the commercial video camera. High quality video cameras can be used to enhance the cardiovascular signal [45], which makes the overall system costly. Moreover, the signal quality is related to the size of the region of interest (ROI). The larger the ROI, the better the quality of signal produced. However, the computation becomes more complex when the ROI is large (i.e., the whole human face), hence a burden for low computing resources devices such as smart phones [46].

Breath rate generated from ECG and PPG is obtained from the breath modulation in amplitude and frequency of the heart signal and therefore is approximate. A more accurate method of acquiring BR can be done by sensing the breath air components, temperature and humidity. Next, we will discuss vital signs detection using breath air-sensing methods.

### 2.3. Air Components-, Temperature- and Humidity-Based Methods

Human breath rate can be measured using characteristics of the inhaled and exhaled air such as its carbon dioxide (CO_2_), its humidity and temperature.

#### 2.3.1. Air Components

**Figure 3:**
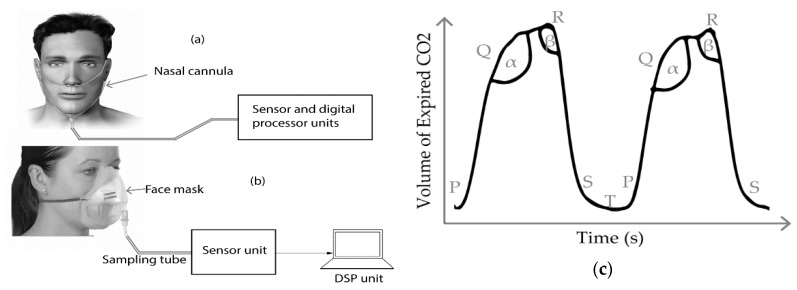
CO_2_ measurement techniques: (**a**) sidestream; (**b**) mainstream; (**c**) description of a capnogram [47].

The level of CO_2_ contained in the air we breathe differs from inhalation to exhalation. Typically, the inhaled air contains approximately 0.04% of carbon dioxide while the exhaled air contains around 6% [34]. This difference in CO_2_ levels can be measured using chemical sensors to determine the breath rate. The most common sensors used are the infrared sensor and the fiber optic sensor, although the former is more popular than the latter. The technique used to determine human breath rate from CO_2_ level variations between inhaled and exhaled air is called capnography. A capnography system is mainly composed of a CO_2_ sensor, gas sampling tube and a signal acquisition and processing unit. The setups for CO_2_ level measurement are depicted in Figure 3a,b. Two main capnography setup methods are available: the side stream method and the mainstream method [24]. In the side stream measurement technique, the sensor and the main processing units are placed away from the subject. In contrast to the side stream setup, the chemical sensor in the mainstream method is located between the processing unit and the endotracheal tube, which is attached to the facial area of the subject as seen in Figure 3b. The mainstream acquisition technique is faster and more accurate than the side stream one. However, it is more expensive and its sensor heats up about 40 °C, which may be damaging to the skin of the subject [44,45].

The waveform obtained from a capnography is called a capnogram. A typical capnogram has three different phases, namely the inspiration, alveolar and expiration phases as seen in Figure 3c [47]. The expiration, alveolar and beginning of inspiration are denoted by PQ, QR and RS, respectively, while the latency phase is represented by ST. The angles α and β indicate transition between respective phases.

Despite their insensitivity to the motion artifact of the subject, breath rate measurement based on capnography is quite uncomfortable for long-term monitoring and can be very sensitive to other gas components and changes in humidity and temperature of the environment [36].

#### 2.3.2. Air Temperature

**Figure 4:**
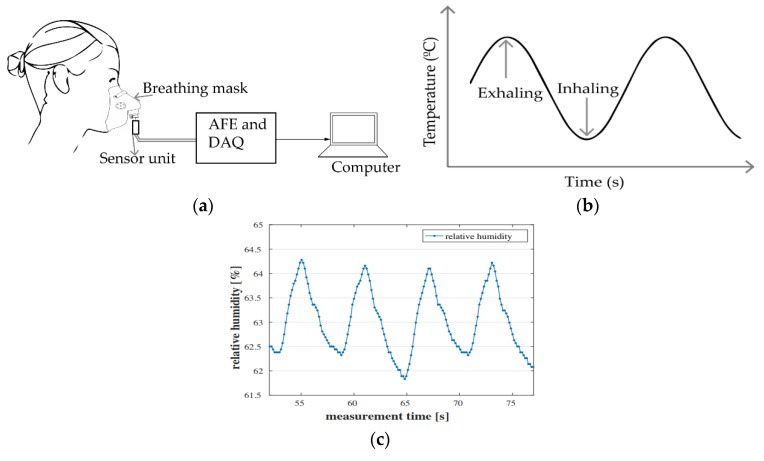
(**a**) General setup for breath airflow-based vital signs monitoring system; (**b**) time response of a thermistor sensor for breath rate (BR) acquisition; (**c**) humidity variation with time [59].

The temperature of the inhaled and exhaled air are different (i.e., the exhaled air is warmer than the inhaled one). This difference can be as high as 15 °C [48]. The air temperature-based breath rate measurement is based on acquiring the temperature variation between the inhaled and the exhaled air. A temperature sensor is used to sense the temperature of breath air of the subject and convert it into electrical signal (e.g., voltage or current). The signal is enhanced by an analog interface before being processed to obtain the breath rate (Figure 4a). Different types of transducers are used to convert the temperature of the airflow into an electrical signal. These includes, but not limited to thermistors, thermocouples, pyroelectric sensors and fiber optic sensors.

Thermistors are resistors with resistance non-linearly dependent on temperature [49]. Thermocouples are thermoelectric sensors producing a voltage signal due to the temperature difference between two conductors (Seebeck effect) [50]. Breath rate detection using thermistors and thermocouples is accurate and relatively cheaper. On the other hand, pyroelectric sensors are based on the production of electric current when the sensor interface is heated by the exhaled air [51]. For breath monitoring, the sensor maybe embedded in a face mask or a headphone [52]. Breath rate acquisition using pyroelectric sensors has comparable performance with the aforementioned thermistors and thermocouples-based monitoring. Moreover, sensors based on fiber optics are employed in many recent works [53,54]. These sensors are based on the shift in Bragg wavelength due to temperature variation of the airflow [53]. Even though fiber optic sensors are more expensive compared to the thermistors, they provide faster response. Nevertheless, their application is limited to clinical settings due to their bulky size.

Figure 4b shows the response of a typical air temperature-based breath rate monitoring using thermistor sensors. It resembles a sine wave depicting the increase and decrease in the airflow temperature during respiration. Breath rate monitoring systems using air temperature variation are simpler and cost effective in general. However, their applications are limited to simple breath rate detection and do not provide heart rate.

#### 2.3.3. Air Humidity

Similar to the capnography method previously discussed, breath rate can be measured by acquiring the water vapor level of the inhaled and exhaled air. The related humidity of the inhaled air differs from that of the exhaled one by approximately 20% to 60% [54]. This difference in humidity can be acquired and plotted indicating the instantaneous respiration pattern. The general representation of air humidity-based breath rate detection systems is depicted in Figure 4a. The sensor in question is a humidity sensor, which can be of capacitive or resistive types. The capacitive and resistive sensors change the capacitance and resistance values, respectively, due to exposure to humidity. This change in capacitance or resistance can be measured and related to the inhaling and exhaling of air. Other types of sensor including the sensors using nanoparticles [55], nanocomposite [56], fiber Bragg grating (FBG) [57] and surface acoustic wave (SAW) [58] can be used as humidity sensors. However, monitoring systems using nanocomposites and nanoparticles have relatively slower response time [56]. Figure 4c depicts the instantaneous change of breath air humidity from a human subject. The graph was extracted from [59].

Like the aforementioned air-based vital sign detection and monitoring systems, the breath rate detection systems employing humidity sensors require the subject to wear a face mask or a tube around the nostril to avoid the corruption of the respiration signal by MAs. As we will see in the next section, vital signs measurement using chest displacement sensing can be non-invasive while providing good accuracy.

### 2.4. Chest-Wall Mechanical Displacement Sensing and Blood Pressure-Sensing Methods

Human breath and heart rates can be measured by sensing the mechanical or physical activities of the heart and lungs at the surface of the body.

#### 2.4.1. Chest-Wall Displacement Sensing

During normal human respiration, the diaphragm contracts and expends along with the intercostal muscles, allowing air to enter and exit the lungs. These physical activities of the diaphragm and respiratory muscles result in displacement of the chest that can expand up to 7.37 cm circumferentially [60]. Different types of sensor can be used to detect the physical movement of the chest. Nevertheless, the most common methods of measuring the chest-wall displacement are based on strain sensing, transthoracic impedance sensing or impedance pneumography and movement sensing using accelerometers, gyroscopes, and magnetometers.

**Figure 5:**
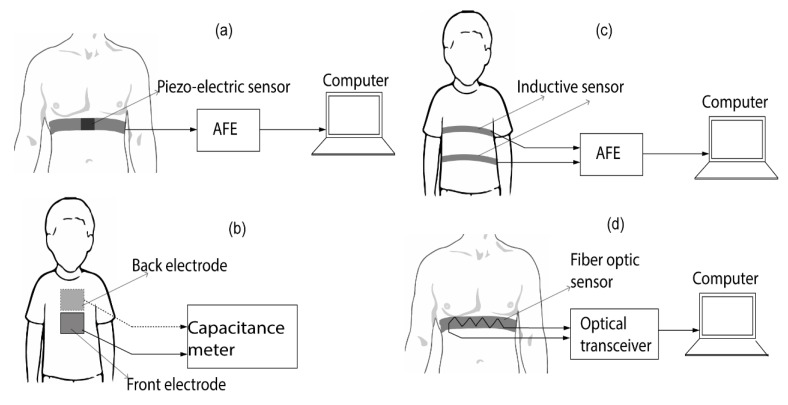
Strain-based sensing methods of breath rate: (**a**) resistive sensing, (**b**) capacitive sensing, (**c**) inductive sensing and (**d**) fiber-optic sensing.

The strain-sensing method employs resistive, capacitive, inductive and fiber-optic sensors to record the instantaneous change in strain. Piezo-resistive strain sensors employ elements that change their shape when subjected to a physical displacement. This change in shape results into a change in their resistance values which are measured using an electronic circuit (Figure 5a). The strain-sensing elements in piezo-resistive sensors is often referred to as “strain gauge” [61]. The strain gauge can be of textile with a coated or embedded conductive element [62,63]. Furthermore, capacitive strain sensors use two different electrodes placed opposite to each other around the abdomen of the subject, as depicted in Figure 5b. The capacitance between the two electrodes is measured using a capacitance meter [64]. Since the capacitance is related to the permittivity of the dielectric material between the electrodes as well as their separated distance, the capacitance value of the two electrodes change during inhaling and exhaling of the subject as the lung air changes the permittivity and the expansion and contraction of the thorax changes the distance between the electrodes. Although the electrodes can be flexibly attached to the human body, tightly attached electrodes exhibits better performance in terms of noise and sensitivity [65]. Furthermore, respiratory rate can be estimated using the change of alternating current in a magnetic coil during breathing activities [66]. Called respiratory inductive plethysmography (RIP), this technique uses the alternating current variation in a magnetic coil attached to the abdomen or thorax of the subject to provide the breathing pattern (Figure 5c). The change in volume of the magnetic coil during the respiration changes the inductance value of the coil and hence the alternating current flowing through it which can be measured. RIP systems have been implemented to monitor sleep apnea [67,68] with good results. However, the motion artifact from the thorax of the subject may undermine the validity of the breath signal [66]. Lastly, fiber-optic sensors can be used as a strain-based respiration sensor [69] (Figure 5d). FBG sensors can be embedded in textile [69,70]. These sensors have faster response time and higher sensitivities compared to their resistive, capacitive and inductive counterparts. Due to their higher sensitivity to small mechanical movements, they can be employed to detect heartbeat signals [71]. Strain-based respiration sensing using smart textiles is extensively used in literature to monitor human respiration and for diagnosis of breath-related diseases [72,73,74,75].

**Figure 6:**
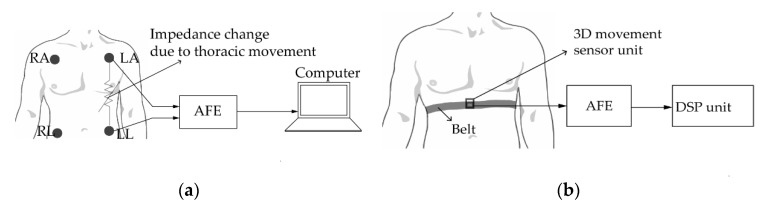
(**a**) Impedance pneumography setup; (**b**) 3D movement sensor setup for breath rate acquisition. The sensor can be an accelerometer, a gyroscope, a magnetometer or a combination of them.

Moreover, the impedance pneumography consists of measuring the impedance of the thorax, which is related to the volume of the lungs. Figure 6a represents the measurement setup of an impedance pneumography. An alternating current (AC) is injected through the skin of the subject using electrodes attached to the chest area, and the voltage difference between electrodes is measured [76]. This voltage difference is proportional to the injected current and the impedance of the thorax. The number of electrodes can be 2 or 4, the latter showing more accuracy [76]. The AC frequency is typically high (about 50 kHz) and the injected current is less than 1 mA. The typical value of thoracic impedance is 500 Ω. This value fluctuates during breathing activities. Transthoracic impedance measurement methods have been employed in monitoring sleep apnea [77], identifying childhood pneumonia [78] and monitoring breath rate during exercise [79]. Even though the breath rate acquisition from impedance pneumography systems are proven to be accurate and do not require tight attachment of sensors to the body of the subject unlike the strain-based sensing methods, they suffer from noise resulting from MA.

Finally, respiratory rate can be detected by measuring the acceleration, the angular velocity and the magnetic field strength of the abdomen during respiration. An accelerometer can be used to convert the mechanical movement of the abdomen into an electrical signal. The movement of the thorax is related to the inertial response of the accelerometer. By attaching this electromechanical device to the upper thorax, it is possible to record the human breathing pattern [80,81]. The measurement results from a triaxial accelerometer is shown to be more accurate in any body orientation, unlike the single and dual-axis accelerometers [82]. Despite the lack of intensive research on breath monitoring using accelerometers [82], this type of system suffers from high measurement errors during exercise and walking activities [83]. Furthermore, a micro-electromechanical system (MEMS) gyroscope can be used to estimate breath rate by measuring the angular velocity of the thorax during respiration. An insight into the working principle of gyroscopes can be found in [84]. As gyroscopes can only determine rotational movement of the thorax, any drift in their three-dimensional (3D) position can result in errors [85] in breath rate detection. Therefore, they are often used in conjunction with accelerometers to provide more accurate signals [86]. Furthermore, movements from breathing activities can be detected by measuring the magnetic field strength around the chest area using a magnetometer. The magnetic field variation can be measured by either simply placing a magnetometer on the chest of the subject using a belt [87] or placing a magnet and a magnetometer on the back and chest area of the subject, respectively [88]. Nevertheless, the use of a magnet enables lower power consumption. Magnetometers function best for quiet respiration, as the sensor data can be highly corrupted by motion artifacts. On the other hand, a sensor unit can be formed by incorporating a triaxial accelerometer, a gyroscope and a magnetometer to monitor the 3D movement of the thorax. Called the inertial movement unit, this new sensor is proven to be less sensitive to motion artifacts [89] and, therefore, can provide better respiration data. The standard setup of a 3D movement sensing-based respiratory rate monitoring system can be seen in Figure 6b.

#### 2.4.2. Blood Pressure Sensing

**Figure 7:**
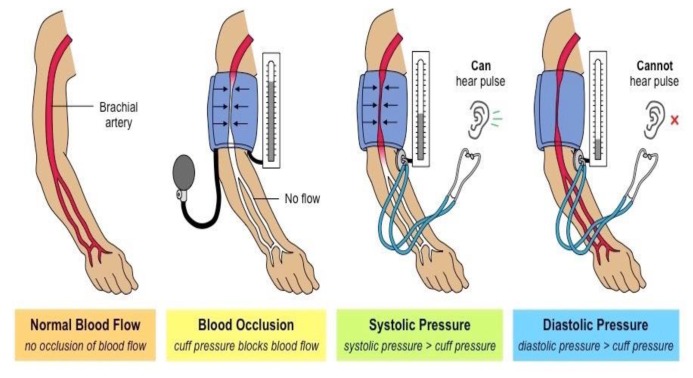
Systolic and diastolic blood pressure (BP) measurement using cuff [101].

Heart rate can be monitored by sensing blood pressure. The rhythmic pulsation of the heart causes pressure of blood on the walls of blood vessels. A sensor can be appropriately placed on the human body (e.g., wrist, upper arm) to capture the instantaneous pressure of blood resulting from heartbeat [90]. The blood pressure measurement can be either non-invasive or invasive. The non-invasive methods of measuring blood pressure include palpatory, auscultatory, ultrasonic, tonometry and oscillometric sensing [91]. The palpatory and auscultatory methods are manually performed by the physician. In the palpatory method, an inflatable cuff is placed around the upper arm of the subject [92]. The health specialist applies pressure on the cuff until there is no blood flow in the branchial artery. A manometer connected to the inflatable cuff is used to display the pressure applied. The systolic pressure of the subject corresponds to that resulting from the pulse while the cuff is under pressure. Even though the palpatory sensing of blood pressure is less dependent on environmental factors, it can only measure the systolic blood pressure of the patient [92]. Similarly, in the auscultatory method, the taping sounds or Korotkoff sounds are detected by a stethoscope which is connected to the cuff. The physician slowly applies pressure on the inflatable cuff attached to the upper arm of the patient. The onset sound detected by the stethoscope represents the systolic arterial pressure of the patient while the last sound detected corresponds to his diastolic pressure. Despite its ability to measure both the systolic and the diastolic blood pressures, this intermittent method is not suitable for continuous monitoring of vital signs. The oscillometric technique on the other hand can be automated even if it is not used for continuous measurements. In this technique, the cuff is inflated to a preset value. While the pressure is being released, oscillations appear and disappear at blood vessel. These oscillations are recorded by the manometer. The maximum oscillation is equivalent to the mean arterial pressure. Different algorithms can be used by different manufacturers in order to determine the systolic and diastolic blood pressures [93]. However, in principle, the systolic blood pressure corresponds to the increase in oscillation when blood flows through the cuff while the diastolic pressure corresponds to the disappearance of the oscillations [93]. It is reported in [94] that the oscillometric method had similar blood pressure measurement accuracy as the auscultatory method. Furthermore, ultrasonic sensors or flowmeters can be used to measure the systolic and diastolic blood pressure. This type of measurement is based on the Doppler effect where the ultrasonic frequency shift due to blood movement is proportional to the velocity of the blood flow in the vessel. The sensor can, therefore, capture the blood pressure in the form of an image. The ultrasonic measurement of blood pressure usually involves a cuff [95] and the sensor is often placed on the patient’s carotid artery [96,97,98]. Furthermore, the arterial tonometry requires a manometer sensor placed on top of the radial artery around the wrist. The force exerted by the sensor flattens the artery and hence the intra-arterial blood pressure is directly transmitted to the manometer through the skin [99]. The use of inflatable cuff is not necessary in the arterial tonometry blood pressure measurement. Despite its ability to continuously measure the blood pressure in an automated manner, this method suffers from inaccuracies due to misalignment, vertical and moderate pressurizations [100]. Figure 7 [101] depicts the general system setup of a cuff-based blood pressure measurement technique. Alternatively, blood pressure can be continuously monitored using invasive methods. For instance, a needle attached to a tube called a cannula can be inserted into the artery of the patient to measure blood pressure through a manometer. This method gives the most accurate results of blood pressure and hence heart rate. However, it is not practically desired for long durations monitoring due to its invasive nature. The aforementioned methods are primarily used to measure blood pressure even though heart rate and heart rate variability can be extracted as well. A method used to obtain solely heart signals is phonocardiography (PCG), which will be discussed in the next section.

### 2.5. Phonocardiography (PCG)

**Figure 8:**
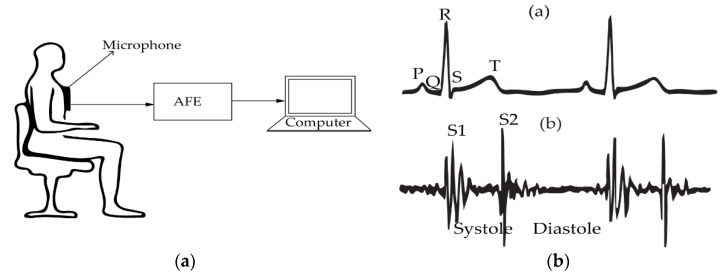
(**a**) Measurement setup of cardiac sound; (**b**) comparison of phonocardiography (PCG) and ECG signals.

During the opening and closing of the valves, the heart produces sounds that can be detected by a microphone. The production of this acoustic effect results from several actions including contraction and relaxation of the heart muscle, rising and falling pressures of cardiac cavities, opening and closing of the valves and blood circulating and stopping. The record of this cardiac acoustic phenomena may help cardiac events to be visualized [102]. The system involving the detection of the heart rate based on the cardiac sounds is called phonocardiography (PCG). The system setup of PCG is given in Figure 8a. It is composed of a transducer element (i.e., a microphone), an analog interface containing an amplifier and a filter and a signal processing unit. The transducer can be attached to the patient’s body using a belt or embedded into a cloth. Figure 8b represents a typical heart sound sketch in comparison with the gold standard ECG signal. Due to the cardiac mechanical activities, two normal sounds denoted by S1 and S2 can be recorded. In general, S1 has lower frequency compared to S2. The systole cycle is indicated by the time taken by the heart to leave S1 and arrive at S2, while the diastole is the duration between S2 and S1. There are two abnormal sounds (S3 and S4). The abnormal sounds are often referred to as murmurs. Murmurs and other abnormal cardiac sounds can be indicators of disease [103]. The PCG curves demonstrate quasi-accurate beat-to-beat cardiac pattern and is comparable to the ECG acquisition system. Despite its wide applications in the early twentieth century, PCG failed to keep up with standardization due to the emergence of other auscultatory cardiac signal assessment methods [104]. This is also due to the vulnerability of the system to surrounding acoustic effects and sounds from respiration that are much stronger than the cardiac sounds.

Table 1 represents the summary of different state-of-art contact-based cardiorespiratory rates measurement techniques. ECG and PPG systems are capable of detecting both the HR and BR. On the other hand, the breath air-based sensing and chest mechanical displacement sensing methods are mainly capable of detecting BR, while BP sensing and PCG can detect mainly the HR. As seen from the table, the motion artifact appears to be one of the main challenges of contact-based BR and HR measurement methods. As previously discussed, these techniques involve the use of contact electrodes. Often, these probes need to be tightly attached to the body of the patient in order to obtain more accurate results. Therefore, they may cause skin irritation and discomfort, making them impractical for long-term or continuous monitoring. Therefore, many recent research activities were directed towards the development of contactless cardio-respiratory monitoring systems using radar techniques. The following section will be devoted to the application of different types of radars in vital signs detection.

## 3. Contactless Vital Signs Detection Using Radar Techniques

Unlike the aforementioned contact-based vital signs monitoring systems, radars do not require any contact probe to be attached to the body of the human being to acquire respiratory and cardiac rates with high accuracy. Vital signs detection radars rely on the modulation effect of a radio signal sent by a transceiver towards the patient. This modulation is due to the chest-wall displacement of the patient, which contains both the respiratory and heart signal along with environmental and electronic noises. The noise is removed during signal processing to provide clean vital signs of the subject. Note that the vital signs radars do not have severe power emission requirements for short distances application (up to a couple of meters). Typically, the power transmitted by the radar does not exceed 12 dBm for a two-meters distance application, which is less than the average power emitted by a smartphone. Therefore, these radars are safe. Depending on the type of signal it transmits, the radar can be of type continuous-wave (CW), pulsed, frequency-modulated continuous-wave (FMCW) or stepped-frequency continuous-wave (SFCW).

### 3.1. Continuous-Wave Radars

#### 3.1.1. Operation Principle

**Figure 9:**
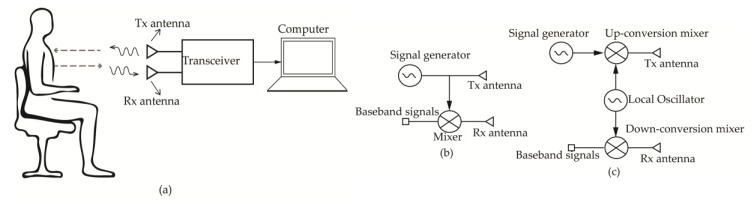
(**a**) Block diagram of vital signs Doppler. Basic transceiver architectures: (**b**) zero intermediate frequency (IF); (c) heterodyne.

The CW radars are the most common type due to their simplicity. A typical radar has a transceiver unit connected to the transmitting and receiving antennas and a digital signal-processing unit, as seen in Figure 9a. The transceiver sends a single-tone continuous-wave signal towards the moving chest of the subject through the transmitter (Tx) antenna. The reflected wave is captured by the receiver (Rx) antenna. The received signal is demodulated and processed by a computer to obtain the breath and heart rates.

The time-domain transmitted signal, denoted by T(t) and the received signal by R(t) are:

(1) $$T\left(t\right)=A_{t}\mathrm{Cos}\left(\omega t+\varphi \left(t\right)\right),$$

(2) $$R\left(t\right)=A_{r}\mathrm{Cos}\left[\omega t-\frac{4\pi }{\lambda }\left(d_{0}+x\left(t\right)\right)+\varphi \left(t-\frac{2d_{0}}{c}\right)\right],$$

where A_t_ and A_r_ are the amplitudes of the transmitted and received signals, respectively; ω the angular frequency of the transmitted signal; λ the carrier wavelength; c the speed of the light; d_0_ the constant distance between the antennas and the subject; x(t) the instantaneous displacement of the chest, given by (3):

(3) $$x\left(t\right)=A_{b}Cos\left(\omega_{b}t+\varphi_{b}\right)+A_{h}Cos\left(\omega_{h}t+\varphi_{h}\right),$$

where A*_b_*, A*_h_*, φ*_b_* and φ*_h_* are the amplitudes and the phase shifts of the chest displacement due to breathing and heartbeat, respectively. As seen from (1) and (2), the signal sent by the radar is modulated in frequency and phase due to the displacement of the chest. This modulation is called the Doppler effect.

The transceiver can be of type heterodyne, where the intermediate frequency (IF) is non-zero or type zero-IF (direct-conversion), where the IF is zero. As shown in Figure 9b, the zero-IF transceivers are simpler and thus less power hungry than their heterodyne counterparts. However, they suffer from large flicker noise corruption and direct-current (DC) offset [105]. On the other hand, the heterodyne structure often requires both up-conversion and down-conversion of the signal and a local oscillator (LO) (Figure 9c), increasing the power consumption and complexity of the system. Another commonly used CW radar architecture is the low-IF CW radar, which is similar to the heterodyne structure with IF values ranging from a couple of kilohertz to several megahertz [106,107]. This type of CW radar allows not only the suppression of DC offset through filtering but also the use of commercially available analog-to-digital-converters (ADCs) [106]. Moreover, the low-IF radar transmission can be either double sideband (DSB) where the upper and lower intermediate frequency components are transmitted and received, or single sideband (SSB), where only one intermediate frequency component is received [107]. In [107], it has been shown that the SSB transmission mode with quadrature demodulation avoids the null-point detection, which occurs when the received signal is in phase or 180° out-of-phase with respect to the local oscillator frequency [108]. Even though the low-IF transceiver architectures have better performance in terms of noise and DC offsets, the direct-conversion architectures are preferred for millimeter-wave applications due to their simplicity. However, it is difficult to design high performant I/Q mixers at these frequencies. To deal with the DC offset and the RF leakage to the baseband branch, a band pass filter can be placed after the quadrature mixer in Figure 9b.

The baseband signals are obtained after demodulation by the quadrature mixer then subsequently filtered, further amplified by the variable-gain amplifier (VGA) and digitized by the ADC before being processed by a computer. For a zero-IF radar, the in-phase baseband signal B_I_ (*t*) is obtained by mixing a replica of the transmitted signal in (1) with the received signal in (2):

(4) $$B_{I}\left(t\right)=T\left(t\right)\times R\left(t\right),$$

Similarly, the quadrature baseband signal is obtained by mixing the received signal with a replica of the transmitted signal shifted by a phase of 90°:

(5) BQ(t)=T(t−π2ω)×R(t),

The above *I* and *Q* baseband signals are used in signal processing to determine the vital signs.

#### 3.1.2. Algorithms and Signal Processing

Various analysis methods can be used with the data of CW in order to accurately extract the vital signs. These methods can be classified as time-frequency analysis, numerical analysis, classification and training and algorithms based on mathematical and experimental modelling [108].

**Figure 10:**
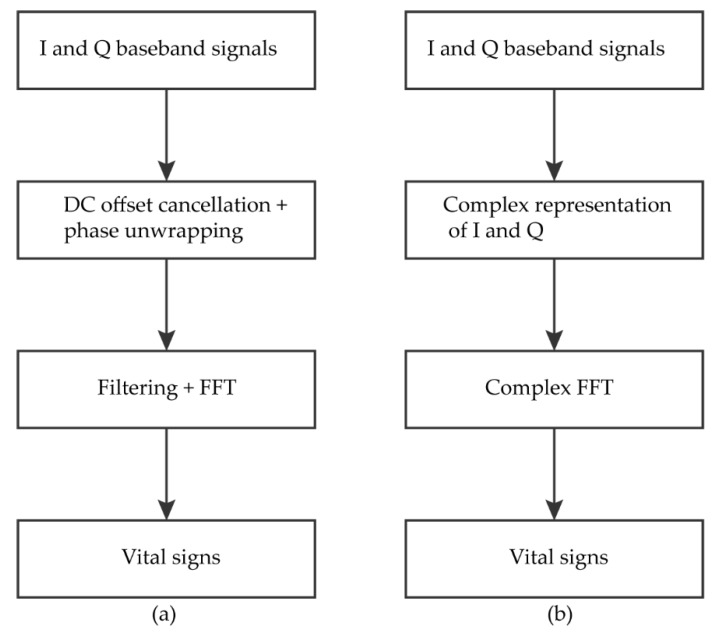
Flow chart of (**a**) arctangent demodulation (AD) signal processing and (**b**) complex signal demodulation (CSD) signal processing.

The time and frequency domain analysis have been extensively used in the literature to obtain HR and BR from CW Doppler radars. Peak detection methods are mainly used as a time-domain method to detect the peaks of respiration and heart signals. These include the autocorrelation output with fast Fourier transform (FFT) [109] and methods based on statistics of the time domain data to differentiate between the time-varying signals and the signal corresponding to stationary objects [110]. Furthermore, the frequency methods use Fourier transform techniques and the chirp Z-transforms (CZT) to extract the vital signs. The FFT is the most popular Fourier transform method used in radar signal processing. The two most common signal analyzing methods exploiting FFT for CW Doppler radars are the arctangent demodulation (AD) and the complex signal demodulation (CSD). In AD, the quadrature baseband signal is divided by the in-phase baseband signal, and the arctangent of the resulting signal is obtained. Furthermore, the FFT is applied to extract the spectrum of the heartbeat and the respiration signals (Figure 10a). A differentiator is often used to the signal before taking the FFT in order to remove the DC offset. The AD method is moderately insensitive to the phase noises of the LO and the mixer, but limited by the DC offset and phase imbalance of the *I* and *Q* baseband signals [108]. On the other hand, the CSD consists of expressing the baseband *I* and *Q* signals into a complex form using Bessel’s function and taking the complex Fourier transform of the result [111] (Figure 10b). The CSD is more robust and tolerant to DC offset, however, it suffers from the harmonics and noise in the baseband signals. On the other hand, the CZT can also be used as an alternative to the FFT [112] and provides good accuracy with a smaller number of samples [113].

Moreover, different numerical analysis approaches can be employed to analyze the CW radar data [114]. These include the mean of signals (MEAN), the least squares (LS), The Hough transformation (HOUGH) and the particle filter (PF). These methods are used to identify the offset component of the data. The MEAN is used to estimate the offset of the signal by assuming the offset remains stable while the phase data changes between 0 and 2π. In the LS method, the data is fit into a circle with center corresponding to the offset. In the HOUGH method, the data is divided into grid cells and the Hough transform is used to define a circle from the grids. Alternatively, a Bayesian filter can be used as PF to estimate the offset of the data. Furthermore, a method called the direct phase estimation based on vector difference can be used to determine the vital signs. Cardio-respiratory rates can also be estimated using the extended Kalman filter (EKF) [115] with fair results. The wavelet analysis is widely used to determine human HR and BR due to its effectiveness for low-frequency signals [116,117].

Furthermore, classification methods can be used to process CW radar data and estimate the BR and HR. In [118], an algorithm was developed for classifying different sleep stages based on the respiration and the body movements. The developed sensor exhibits good results in terms of sleep/wake pattern monitoring. Moreover, a bio-sensor was developed in [119] using a linear discriminants classifier for determining sleep/wake pattern.

Finally, experimental and mathematical modelling can be used to estimate human vital signs. In [120], the authors established a linear relation between lung volume and chest-wall displacement using experimental data. The radar data can, therefore, be used to estimate the tidal volume based on this relationship. Furthermore, the authors in [121] proposed a mathematical model relating the intrapulmonary pressure and the chest-wall displacement. Then, the tidal volume is estimated using the baseband signals of the Doppler radar. The results from these modelling methods show good accuracy.

#### 3.1.3. Biomedical Practice

Doppler CW radars have been used for cardio-respiratory signals sensing since 1975, when Prof. James C. Lin set up an experiment to wirelessly measure the respiration rate of a rabbit and human subjects located at 30 cm to the device [122]. Since then, a lot of research activities have been undertaken to improve the performance of this type of radar. In [109], a CW radar operating at 2.4 GHz was designed and tested with a human subject located at 1.5 cm from the radar. The respiratory and cardiac rates obtained exhibited mean errors less than 0.5 beat/min and 1 beat/min, respectively. A W-band millimeter-wave radar was successfully designed and tested on a human subject in [123]. In [124], a Doppler radar was designed at 60 GHz for short-range detection of human presence as well as vital signs detection. The experimental results show promising performance for occupancy applications. Multi-target vital signs detection were made possible with Doppler CW radar prototypes using multiple beamforming in [125] and [126]. The aforementioned examples show the ability of CW radar to detect the heart and respiration rates of human subjects under motionless testing conditions. However, the real-world application of vital signs detection radars encounter several difficulties that are discussed next.

#### 3.1.4. Challenges

The CW radars face numerous challenges during the detection of heart and respiration rates including null-point detection, signal corruption from the phase noises of the LO and the mixer, random body movement (RBM) of the subject, separation of heartbeat signal from respiration signal, multiple targets detection etc. The null-point detection and phase-noise issues can be adequately dealt with by using a proper CW transceiver architecture (low-IF single SSB) and signal processing (CSD) [111]. However, issues like RBM, accurate heartbeat detection and multiple-subjects detection require more sophisticated architectures and signal processing techniques, increasing the complexity and power consumption of precise CW vital detection radars. These technical challenges are part of the reasons why vital sign radars do not exist in the consumer market to this day [127].

During the acquisition of the vital signs, the subject may often move body parts like hands and legs or his entire body. These unwanted body movements are called random body movements. The signals reflected due to RBMs are stronger than the vital signals, therefore corrupting the latter. Hence, it is necessary to incorporate an RBM cancellation mechanism in the radar. Several methods of RBM mitigation were proposed in the literature. One method of cancelling the RBM is the use of two identical CW transceivers and the CSD [122]. Another method used to cancel RBM effect in vital signs acquisition is the radar implementation with self-injection locking [128]. In [129], the empirical mode decomposition (EMD) signal-processing technique was used to remove motion artifacts from the antenna and the subject. Another signal-processing method was used in [130] to remove the RBM effect on human vital signs. Even though the forward and backward movements are easily cancelled out in these papers, they usually require more complex and power-consuming systems. The above RBM mitigation techniques are detailed in Section 3.5.

Furthermore, the ability of the radar to detect a precise and accurate heart signal is challenging. The frequency of the human heartbeat (1–3 Hz) lies close to that of the respiration (0.1–0.9 Hz). Since the heartbeat signal is much smaller in amplitude compared to the respiration signal, it can easily be corrupted by the harmonics of the latter. Therefore, it is often required to take adequate measures for recovering the heartbeat signals. In [131], a signal-processing method called parameterized demodulation is used to separate the heart signal from the respiration one. A simple direct-conversion radar with very narrow beam dual helical antennas was designed in [132] for automotive application. The time domain measurement results show the heartbeat signal of a human subject. Nevertheless, the antennas are required to be very directional and the chest-wall displacement due to respiration is assumed to be relatively constant. Another method of accurately detecting the small heartbeat signal in the presence of stronger breathing signal is the use of millimeter-wave (mm-wave) frequencies. Since the phase shift due to the displacement of the target is inversely proportional to the carrier wavelength, smaller movements of the chest-wall due to heartbeat may be detected with precision at higher radar carrier frequencies. In [133] and [134], the authors implemented millimeter-wave radars that were able to detect both BR and HR without using sophisticated signal-processing methods.

Moreover, for applications such as surveillance and human presence detection, the Doppler radar must be able to detect the vital signs of multiple human subjects at the same time. Simple CW radars cannot fulfil this functionality since single tone CW signals cannot provide range information [135]. Therefore, the radar signal needs to be frequency-modulated in order to detect multiple subjects. Examples of such radars include the frequency-modulated continuous-wave (FMCW) radars and stepped-frequency continuous-wave radars (SFCW), which will be discussed next.

### 3.2. Frequency-Modulated Continuous-Wave (FMCW) Radars

#### 3.2.1. Operation Principle

**Figure 11:**
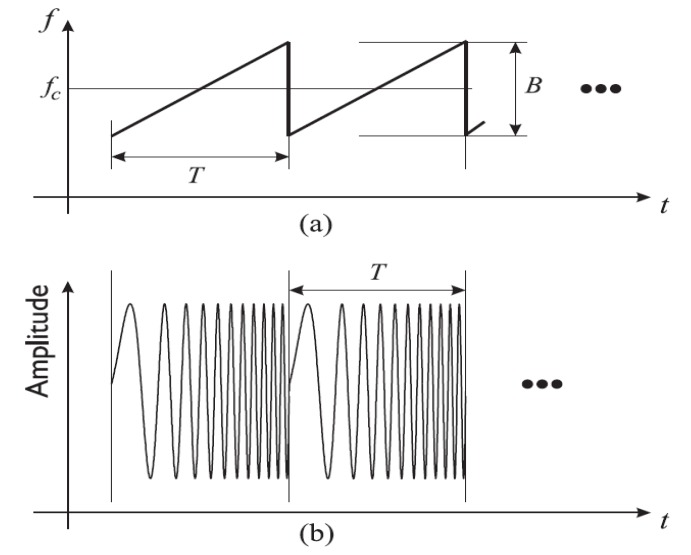
Frequency-modulated continuous-wave (FMCW) signal: (**a**) frequency variation over time, (**b**) instantaneous chirp signals.

In FMCW radar systems, the frequency of the output signal is varied linearly with respect to time. Represented in Figure 11, this type of signal is composed of a unity signal called chirp generated at every period T. The chirp can be generated by feeding a voltage-controlled oscillator (VCO) with a linear control voltage. Alternatively, the chirp can be generated by using a phase-locked-loop (PLL) with frequency synthesizers [136]. The transceiver architectures of FMCW radars are similar to that of CW Doppler ones. Nevertheless, a direct-conversion system is often used in order to decrease the high computational loads [137]. Directly converting the received signal with a replica of the transmitted one is called de-chirping. The demodulated signal is often called the “beat signal”. It contains both the range and micro-doppler information. The data obtained from the radar can be put in a matrix form containing the slow-time and fast time data. The slow-time data is related to the number of transmitted ramps and contains the range information. On the other hand, the fast-time time data indicates the number of samples per ramp and contains the vital signs information.

#### 3.2.2. Algorithms and Signal Processing

**Figure 12:**
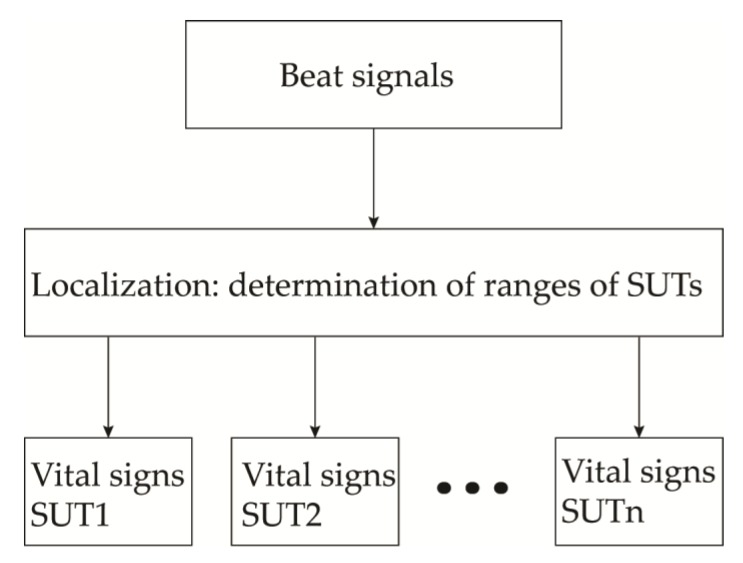
General algorithm used to extract the vital signs of multiple subjects using radar techniques.

**Figure 13:**
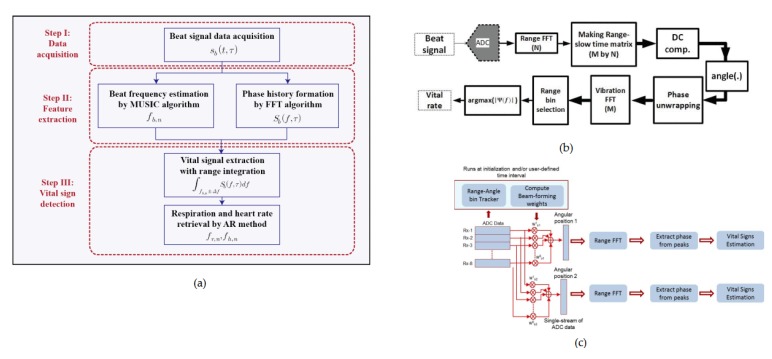
Algorithms used in (**a**) [138], (**b**) [140] and (**c**) [141] to retrieve the range and vital signs of subjects-under-test (SUTs).

The FMCW radars are capable of providing both the range and Doppler information. Therefore, the vital signs of multiple subjects located in the line-of-sight of the antennas can be detected. The basic algorithm of used in FMCW radar data analysis involve localization of the subject and detection of HR and BR associated with each range as seen in Figure 12. Different signal-processing methods can be employed for removing noises, DC offsets or MA effects. However, the most popular signal processing is the time-frequency method employing the FFT to extract the vital signs and the range information. In [138], the authors used an auto-regression algorithm to extract the cardio-respiratory rates of the subjects. In the AR analysis, the regular frequencies are sought among a constant signal, allowing the extraction of HR and BR [139]. The range and the phase information were obtained by the parametric data estimation and the FFT, respectively. Figure 13a shows the summarized algorithm used to detect the vital signs of the subject-under-test (SUT). Furthermore, A series of FFT and DC compensations were used in [140] for determining the vital signs of a patient lying on a bed. The vital signs’ extraction algorithm is given in Figure 13b. In this analysis, the DC compensation is crucial for the phase unwrapping processing, which precedes the retrieval of the vital signs. The results from the experiments show accuracies of 80% and 94% for HR and BR, respectively. Lastly, the authors in [141], uses multiple antennas to detect and locate the vital signs of two SUTs. The block diagram of the signal-processing employed in that work is given in Figure 13c. The frequency domain processing is used to effectively extract both BR and HR. To remove the user-induced motion artifact, a certain signal energy threshold value where any time window signal energy exceeding that value is omitted from the data.

#### 3.2.3. Biomedical Practice

Many recent research on non-contact vital signs sensing are based on FMCW radar techniques as they overcome the shortcoming of the difficulty or inability in CW radars to provide the range information. A portable short-range FMCW radar was developed in [142] for localization and tiny vital signs detection of human subjects. The designed hybrid system was able to differentiate human subjects from other surrounding objects. Furthermore, a millimeter-wave FMCW radar was implemented in [140] for vital signs monitoring of a patient located at around 1.7 m from the radar. Showing relatively low power consumption, this system may be used for long-term monitoring of patients. In [141], An FMCW radar was able to detect a vibrating reflector and a human subject located at the same range bin, with the help of beamforming.

#### 3.2.4. Challenges

The use of FMCW radars for vital signs detection does not eliminate issues such as RBM effect and separation of the respiratory signal from the cardiac signal as previously discussed. Another important issue arising in the operation of FMCW radars is the incoherence of the radar. Incoherence occurs when the radar is unable to detect the micro-doppler signature which is contained in the phase data [137]. Therefore, it is necessary to control the phase of the radar waveform. In addition, the range resolution of FMCW radars is limited by the bandwidth [143]. Higher resolution implies higher bandwidth. For instance, the range resolution of a 10 GHz FMCW radar is around 1.5 cm. However designs of modules such as VCO is challenging due to higher-phase noise level. Furthermore, the VCO may not exhibit a linear frequency sweeping. Hence, calibration is often required in the operation of high-bandwidth FMCW radars [143,144] in order to maintain linearity. FMCW radars provide fine range and Doppler information. However, the power consumption in these radars is relatively higher compared to the CW radars. One type of radar capable of providing both the range and micro-doppler information with relatively lower power consumption is the SFCW radar, which will be discussed in the following section.

### 3.3. Stepped-Frequency Continuous-Wave (SFCW) Radars

#### 3.3.1. Operation Principle

**Figure 14:**
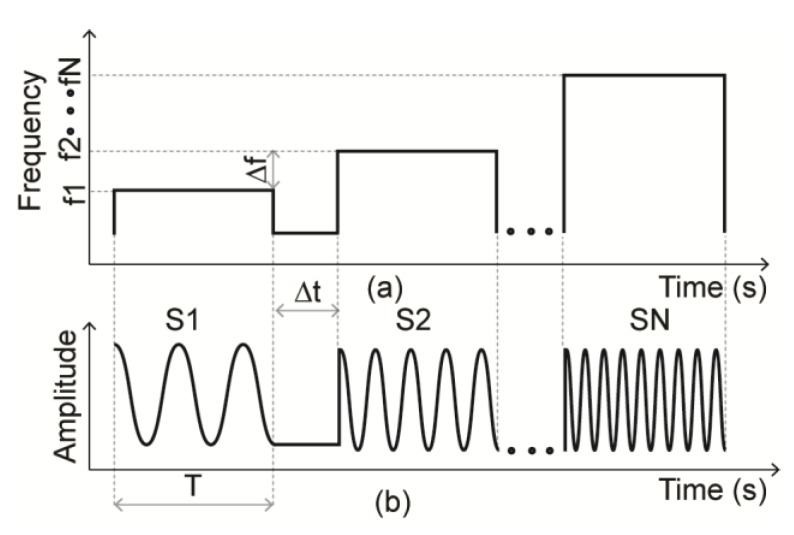
Stepped-frequency continuous-wave radar (SFCW) signal: (**a**) frequency variation over time, (**b**) instantaneous chirp signals.

The operation of SFCW radars is similar to that of FMCW ones. In this case, a series of N frames are linearly transmitted towards the target with a time interval of Δf between each frame, as seen in Figure 14. The reflected signal is down-converted with a replica of the transmitted one to obtain the beat intermediate frequency.

#### 3.3.2. Algorithms and Signal Processing

**Figure 15:**
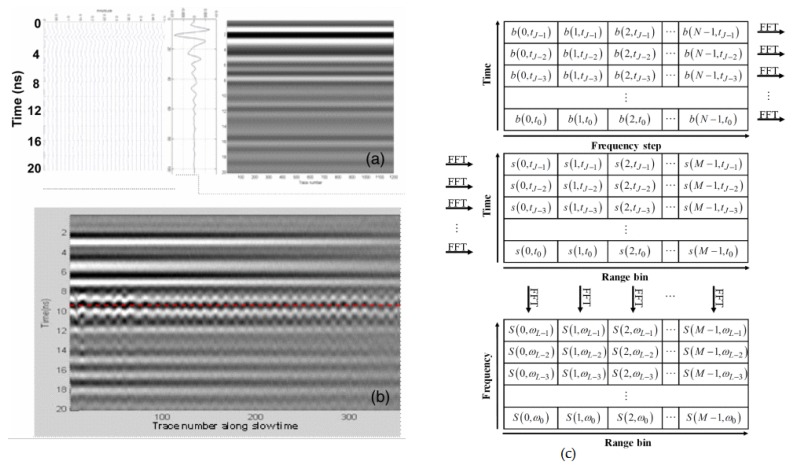
(**a**) Time-domain SFCW radar data with presence of human subject after applying inverse fast Fourier transform (IFFT); (**b**) vital signs after cancelling the clutter effect [146]; (**c**) algorithm used to obtain the range-Doppler profile [145].

The SFCW radars are not common in human vital signs detection applications compared to the CW and FMCW radars. Nevertheless, they detain similar algorithms and signal processing techniques to their FMCW counterparts as they allow localization and multi-subjects detection. Meanwhile, most article on the implementation of SFCW radars for human HR and BR detection use frequency domain processing, peak detection from FFT being the dominant method. The baseband data obtained from the radar is put into a time-frequency matrix of J × N size, where J and N indicate the total number of frequency sweeps and the number of radio frequency (RF) steps, respectively [145]. Next a consecutive FFT is applied to the matrix to obtain a range Doppler profile seen in Figure 15c. An example of raw data obtained after the inverse FFT (IFFT) is applied to the frequency domain signal of an SFCW radar is presented in Figure 15a,b [146]. The radar implemented was used to detect the presence of a human subject under brick layers.

#### 3.3.3. Biomedical Practice

SFCW radars have been actively investigated for biomedical applications. Due to their simplicity compared to the FMCW radar, this type of radar is becoming quite attractive for contactless cardio-respiratory signal detection and monitoring in recent years. In [147], experiments using a SFCW radar was performed to successfully obtain the cardio-respiratory rate and the respective locations of three individuals at the same time. Furthermore, the performance of the radar was evaluated for different parameters including the center frequency of the radar, the bandwidth, the frequency step and the properties of the antenna. An SFCW radar centered at 3 GHz with 2 GHz bandwidth was implemented and tested with a human subject located at different distances from 1 m to 2 m in [148]. The results of the cardiac and respiratory rates showed an error of less than 2%. An experiment was performed to evaluate the performance of an SFCW radar for measuring the vital signs of an individual at different body orientations in [149]. Despite having better performances at the front and back positions, the radar exhibits errors of no more than 2% for all the four positions. In [146], an SFCW radar was setup using a vector network analyzer (VNA) and two antennas to evaluate its detection accuracy of the vital signs of a human under concrete in laboratory conditions. The radar showed capability of non-line of sight detection of human respiratory signals at a distance of 1 m or higher with high speed and regardless of the posture of the subject. This shows a great potential of SFCW radars in the application of search and rescue during natural disasters like earthquakes.

#### 3.3.4. Challenges

Similar to the CW and FMCW radars, the SFCW radars suffer from the motion artifact of the subject as the major bottleneck. Additionally, the presence of respiratory harmonics on the vital signs undermine the accuracy of the heart rate.

Even though the radars discussed in the previous sections have high sensitivity, they suffer from low power efficiency and are not preferred for the detection of humans behind concretes. On the other hand, pulsed radars have the advantage of higher power efficiency and can be used to detect the vital signs of humans located behind non-conductive materials such as concrete walls and tables. This type of radar will be discussed in the following section.

### 3.4. Ultra-Wideband (UWB) Pulse-Based Radars

#### 3.4.1. Operation Principle

**Figure 16:**
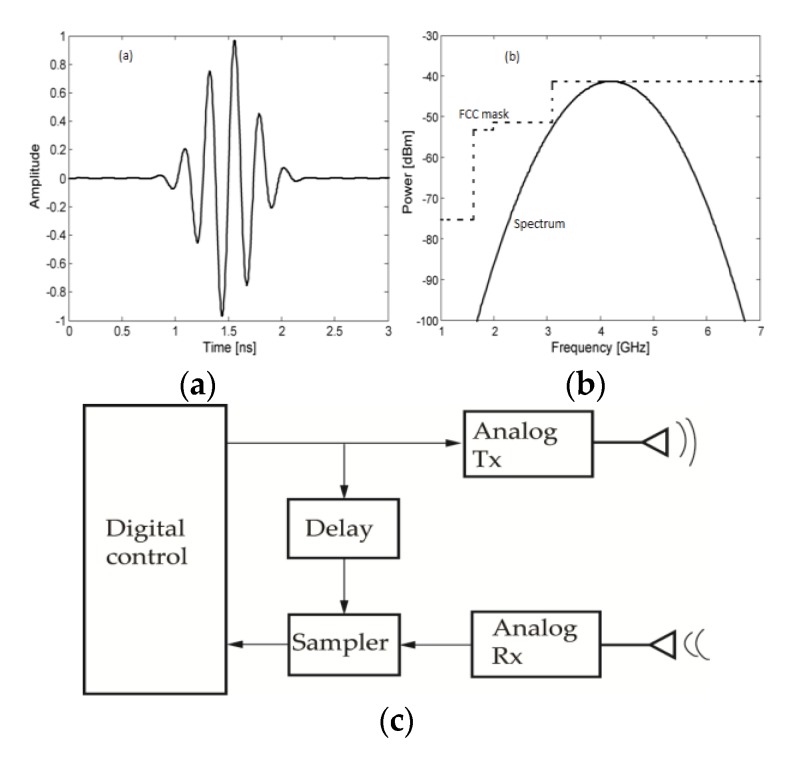
(**a**) Time domain and (**b**) spectrum of an ultra-wideband (UWB) signal; (**c**) basic block diagram of a UWB radar.

In pulse-based sensing radars, a short time (e.g., sub-nanosecond) domain modulated or un-modulated pulse is sent by the radar’s transmitter towards the patient. The reflected echo is captured by the receiver and processed in time domain to obtain the cardio-respiratory signals of the subject. The most common pulse-based radar for vital signs sensing is the impulse radio ultra-wideband radar (IR-UWB). The typical architecture of IR-UWB radars is depicted in Figure 16c. The echoes of the transmitted pulses are received by an analog receiver and sampled using a delayed replica of the transmitted one [150]. The offset block represents a delay equivalent to the time-of-flight of the pulse. The time-of-flight corresponds to the total time it takes the pulse to travel from the transmitter antenna and return back to the receiver antenna. The typical pulse generated by IR-UWB radars is represented in Figure 16a,b.

#### 3.4.2. Algorithms and Signal Processing

**Figure 17:**
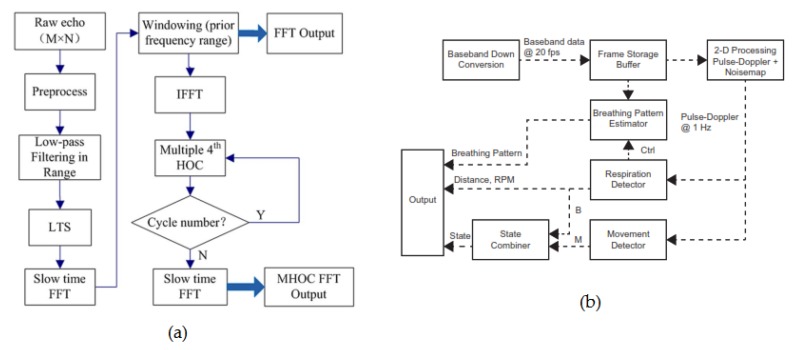
Vital signs retrieval algorithm for the ultra-wideband (UWB) radar in (**a**) [140] and (**b**) [131].

Various algorithms are used to retrieve the cardio-respiratory rates of human subjects using UWB radars depending on the specific applications. A simple UWB employing body movement cancellation was designed in [151]. It uses the CZT method and motion filter in order to retrieve the respiration and eliminate the MA effect. Even though the results appear to be good for detecting the respiration rate, the system designed is not suitable for real-world applications. More sophisticated algorithms are used in UWB radars. In [152], the authors developed a new algorithm based on multiple higher order cumulant (MHOC) and compare the results obtained with an FFT detection method. MHOC results into higher SNR and allows the reduction of high harmonics. Figure 17a presents the flow chart of the new algorithm along with a reference FFT-based algorithm. Moreover, a signal-processing method based on time and pulse-doppler domains were applied in [150] to obtain the cardio-respiratory rates of sleeping individuals with very high accuracy. Figure 17b displays the flow chart of the signal processing. Many other signal-processing methods are used in UWB radars to obtain human vital signs with good accuaracy. Hilbert Huang transform [HHT] and FFT in the time-frequency domain are used to process the received signal in [153]. Classical arctangent demodulation (AD) [154,155,156,157], and complex signal demodulation (CSD) [154,155] are also employed for extraction of vital signs in UWB radars. Clutter effect can be removed by using the singular value decomposition algorithm (SVD) [158]. In [159], the ensemble empirical decomposition (EEMD) and continuous wavelet transform (CWT) were used in a UWB radar to improve the signal-to-noise (SNR) ratio and separate the respiration and heartbeat signals. A low complexity approximation employing the maximum likelihood (ML) estimation of the period of the signal mixed with additive white Gaussian noise (AWGN) was proposed and tested in [160].

#### 3.4.3. Biomedical Practice

The UWB radars are extensively used in many biomedical applications. Due to the ability of the UWB pulse to penetrate concrete, a plethora of research has been conducted into detection of humans behind walls and other concrete objects. This feature gives the advantage of UWB radar for applications such as search and rescue surveillance and security. In [161], a UWB radar and signal processing were developed to detect the human presence behind walls and concrete doors. The results show the capability of the radar to sense humans behind walls and wooden doors. In [162], a ground-penetrating radar based on UWB architecture was implemented and successfully experimented on a human subject under a 3 cm thick table. In [158], a new signal-processing algorithm was developed to remove the clutter noise of stationary and non-stationary object for the detection of human located under a pile of concrete bricks. A monitoring radar was designed and implemented in [163] for indoor localization and fall detection of human subjects. A multiple-antenna radar was designed and tested using different signal-processing methods for real-time localization and tracking of human subjects in an indoor setup in [164]. The radar and signal processing algorithm proposed in [165] was able to successfully detect the vital signs of two stationary persons and a moving person. In [136], a study has been conducted to determine the heart rate variability of human subjects and extrapolate this data to predict the mental state of the subject. The results show an accuracy of 82% for the classification of the mental state.

#### 3.4.4. Challenges

**Figure 18:**
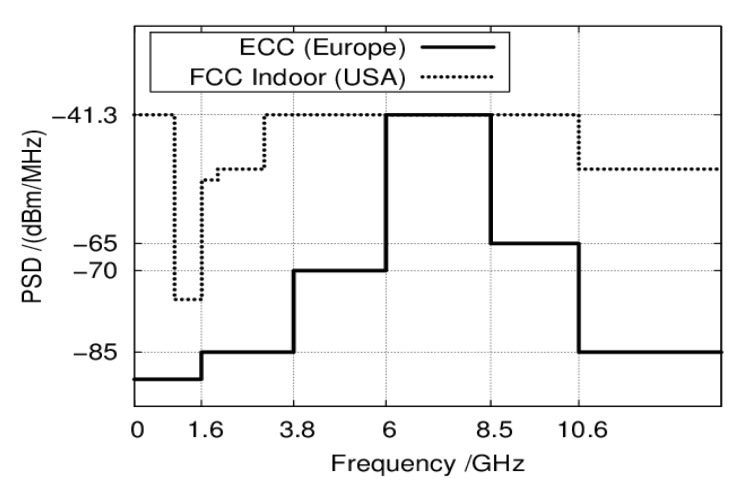
Federal Communication Commission (FCC) and European Electronic Communication Commission (ECC) masks for UWB application [166].

The IR-UWB radars have been proven to have great feasibility in search and rescue and security applications due to their low power consumption and excellent ability to penetrate non-conductive material. However, a number of limitations impede their popularity over the Doppler radars. One of the fundamental issues with the IR-UWB radars is their power density limitation. For safety and interference with other devices purposes, power density masks are provided by telecommunication regulators such as the Federal Communication Commission (FCC) and the European Electronic Communication Commission (ECC) (Figure 18). These masks provide the maximum power density that a UWB device can have. For instance, the power emission in UWB radars is limited to −41.3 dBm/Hz for indoor applications in the United States. As a result, the UWB vital signs radars are limited to short distance applications only. Furthermore, the SNR is high as emitted signal power is low.

Table 2 gives a summary of performance factors of different radar types used for vital signs detection. Meanwhile, the main issue of these radars is the motion artifact effect on the vital signals, which will be discussed next.

### 3.5. Random Body Movement (RBM) Cancellation Techniques in Doppler Radars

As stated in the previous sections, RBM remains the main challenge in human vital signs detection using radar techniques. Several RBM mitigation techniques are studied in the literature.

**Figure 19:**
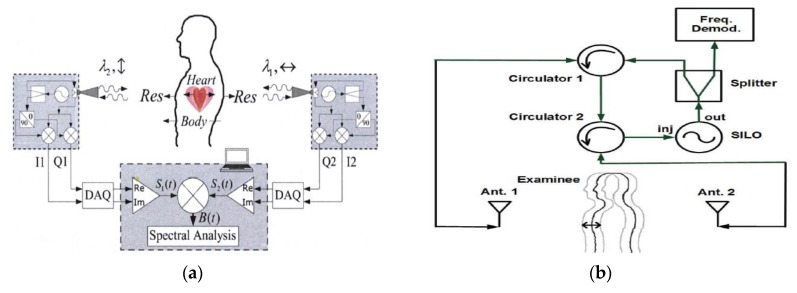
(**a**) Random body movement (RBM) cancellation using two identical transceivers [111]; (**b**) self-injection radar for RBM cancellation [128].

One method of cancelling the RBM is the use of two identical CW transceivers and the CSD [111]. The two transceivers, one placed in front of the subject and the other at the back, simultaneously send a CW signal to the subject (Figure 19a). The respiration and heartbeat of the subject are in-phase for the two transceivers while the velocity of the body movement are opposite. Taking the scalar product of the signals received by the two transceivers, the RBM effect is removed. This technique is able to recover the vital signs of a human subject in the presence of large-scale forward and backward body movements. However, it requires the use of two transceivers that need to be identical and able to send the same types of signals at the same time.

Another method used to cancel the RBM effect in vital signs acquisition is the radar implementation with self-injection locking [128]. As shown in Figure 19b, the radar has two antennas, one located at the front and the other located at the back of the individual. A CW signal is sent from the front antenna towards the chest of the subject. The same antenna collects the received signal and resends it through the circulators and the back antenna towards the back of the subject. The reflected signal from the back of the subject is collected by the back antenna and injected to a self-injection locked oscillator (SILO). As the reflection coefficient of the RBM seen from the front of the subject is opposite to that seen from the back, the signal received by the back antenna will not feature the RBM effect. Even though large-scale body movements are cancelled, the radar requires extra couplers, phase shifters and calibration to remove the reflections from the surrounding environment, rendering the system more complex.

Furthermore, signal processing techniques can be used to remove unwanted body movement in Doppler radars for vital signs detection. In [129], the empirical mode decomposition (EMD) technique was used to remove motion artifacts from the antenna and the subject. In [130] the authors demonstrated the possibility of measuring vital signs of humans by apply the cyclostatic transformation to Doppler radars in presence of RBM. However, the SNR of the received signal must be high enough so the vital signals would not fade during the non-linear transformation.

### 3.6. Heart Rate Variability Assessment Using Vital Signs Radar

The study on vital radars in this paper was mainly focused on the detection of HR and BR and, therefore, does not address the beat-to-beat cardiac activities in details. Heart rate variability (HRV) provides the time variation in beat-to-beat interval and is particularly important for correlating the cardiovascular regulatory system response to stress, illness and demands [167]. In addition to stress and emotion recognition, HRV assessment can be important for anxiety treatment and vigilance monitoring [168]. Doppler radars have the capability of detecting beat-to-beat signal of human cardiac activities with acceptable accuracy. In fact, many recent vitals radars articles were directed to the evaluation of the radar in HRV assessment.

**Figure 20:**
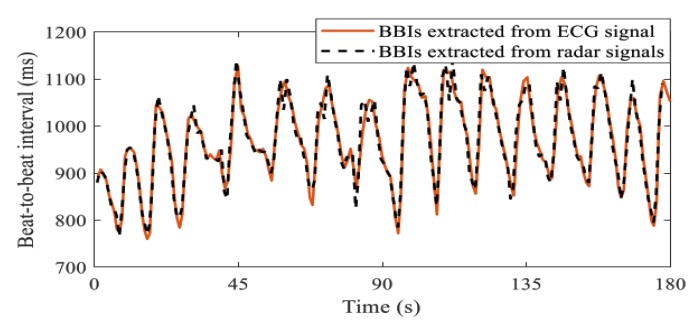
Beat-to-beat intervals (BBIs) obtained from one subject in comparison with an ECG reference [172].

In [169], the authors conducted an experimental study of a 2.4 GHz CW radar in measuring the HRV of three different subjects. The linear demodulation method was used for signal processing of the quadrature data obtained from the radar. The data from a finger pulse reference was also used in comparison with the obtained results. The results obtained from the radar showed high accuracy in detecting beat-to-beat interval as small as 1 ms. Furthermore, the authors in [168] performed the HRV measurement experiment on 10 healthy subjects using a 24 GHz Doppler radar and novel algorithm. The measurement results show a maximum mean relative error of 2.03% for the beat-to-beat interval (BBI) acquisition. Figure 20 displays the BBI measurement obtained from the CW radar in comparison with the measurement results from a reference ECG. Moreover, a millimeter-wave FMCW radar was used in [170] to assess the HR variation of 3 different individuals sitting at distances between 0.6 m and 1 m. A reference measurement reading from Apple Watch show a high degree of agreement with the results obtained. Lastly, a UWB radar was used in [171] for classifying the mental states of different individuals into 4 categories, namely, the normal state, the fatigue state, the stress state and the sleep state using their HRV data. The radar operates at a center frequency of 7.29 GHz. A classification accuracy as high as 82% was obtained, which is sufficient for mental state classification.

### 3.7. Effect of Frequency on the Detection Accuracy of Vital Signs Radars

The effect of frequency on detection accuracy of human vital signs using Doppler radar has not been intensively studied in the literature. However, several factors will need to be taken into account to fully understand how the operating frequency of the radar impact vital signs acquisition. First, the dielectric properties of the human body tissue must be studied. The complex dielectric properties of human tissue was modelled by Gabriel et al. in [172] using the dispersed Cole–Cole equation. It appears that the permittivity of human body tissue is related to the frequency and the conductivity of the tissue. The attenuation coefficient and the complex permittivity of human tissue were estimated for the frequency range 8–18 GHz in [173] and for the range 10–60 GHz in [174]. The real and imaginary parts of the complex dielectric constant decrease at increasing frequencies within the millimeter-wave band. However, the attenuation constant increases and hence, the skin depth decreases. For instance, the skin depth of human skin at 10 GHz is just 2.7 mm [175]. Therefore, the millimeter-wave signal is almost partially reflected and partially absorbed by the human skin, which is the first element to be in contact with the transmitted signal from the radar. Hence, the wave reflection from body tissue at higher frequency is stronger than that at lower frequencies, which is an advantage of using the millimeter-wave frequencies for vital signs detection. Nevertheless, the power level needs to be low enough to avoid strong radiation against human body. Vital signs radar are meant to operate for short range applications (from 0.5 m to 2 m in general), eliminating strict radiated power requirements. Next, the path loss is another effect that plays in the strength of the received radar signal. The path loss is defined as the attenuation of the electromagnetic wave as it propagates through space. Since the spatial attenuation is proportional to the frequency, higher frequencies experience more loss than lower frequencies. However, due the short-distance application and higher sensitivities of Doppler radars, the path loss effect does not significantly affect the accuracy of the radar. Lastly, as mentioned in Section 3.1.3, the phase shift due to chest displacement is proportional to the carrier frequency. Therefore, smaller displacements can be detected when the radar is operating at higher frequencies, which is useful for the detection of heartbeat. However, the detection accuracy of the respiration becomes poor when the frequency is too high [133] because of the introduction of phase ambiguity at target displacement larger than half of the wavelength of the operating frequency (λ/2). Therefore there exists an optimal frequency of operation for the radar. In [176], the authors demonstrated through simulation that the optimal carrier frequency of vital signs Doppler radars for typical chest displacement corresponds to the lower Ka band (e.g., 27 GHz). Furthermore, the harmonics of the respiration become stronger at higher frequencies [177]. Hence, a proper harmonic cancellation mechanism must be employed during the signal processing especially when the CSD is used.

Next, the results from an experiment setup using a CW radar and two human subjects will be presented and discussed. Future works needed for the application of vital signs radars in the consumer market will be emphasized. Next, we will evaluate the performance of a CW radar in respiration and heart rates acquisition using two human volunteers.

## 4. Experiment for Measuring Human Cardio-Respiratory Rates Using a Continuous-Wave (CW) Doppler Radar

### 4.1. Experiment Setup

**Figure 21:**
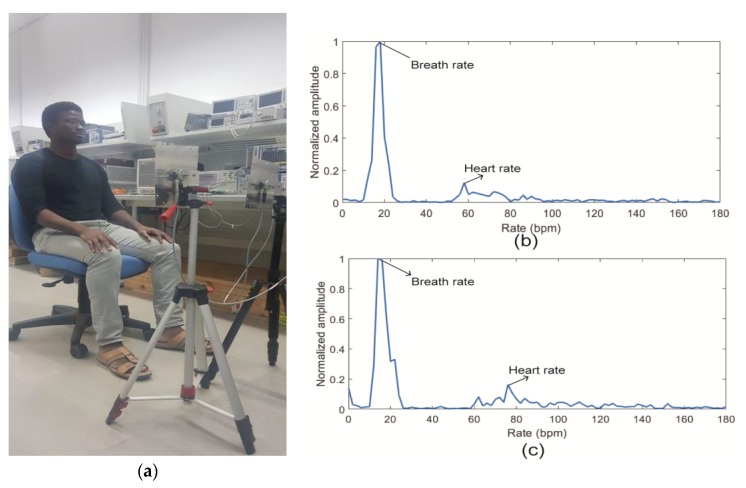
(**a**) Setup of the vector network analyzer (VNA)-based continuous-wave (CW) radar for vital signs measurement; BR and heart rate (HR) of (**b**) Male volunteer and (**c**) female volunteer.

A proof-of-concept experimental setup has been constructed using a vector network analyzer (VNA) and two horn antennas operating from 8 GHz to 15 GHz. As seen in Figure 21a, the SUT sits at a distance of 1 m away from transmitting and receiving antennas, which are directly connected to the VNA. Tripods are used to adjust the elevation of the antennas to the chest of the subject. Single-tone CW at 10 GHz is transmitted and received as carrier. Three different types of experiment were performed with each SUT: the empty measurement, normal breathing, and holding breath. First, empty measurement was conducted when no SUT or moving element is present in the antennas’ line of sight. This type of measurement was performed once. It is used as a reference measurement to perform background subtraction in order to eliminate the clutter effect. In the second type of experiment, the subject breathes normally and quietly with no other body movement. This type was performed at least twice to ensure the agreement of the results. Finally, the subjects hold their breaths while sitting quietly on the chair. This data provides only the heart rate which is compared to the rate obtained when the SUT breathes normally. This type of measurement was performed a couple of times as well. Each experiment session was performed for a duration of 30 s, which is enough to detect the respiration and heart rates. To verify the feasibility of the system, male and a female volunteers participate in this experiment. The signal processing was performed with a laptop using MATLAB software. De-noising, filtering and CSD algorithms were used to obtain the vital signs.

### 4.2. Data Processing

**Figure 22:**
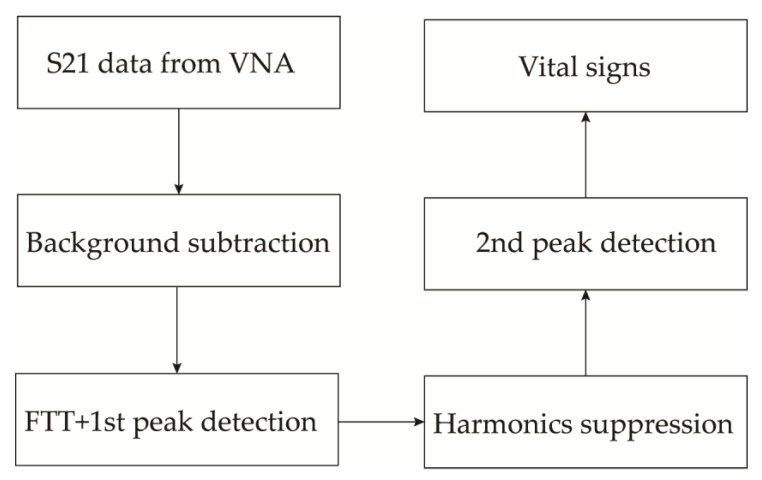
Algorithm used to process the experimental data.

The aim of the signal processing is to obtain the BR and HR of the subject without employing much complex algorithms. The summary of the signal analysis algorithm employed in this experiment is depicted in Figure 22 The first step was to subtract the clutter noise using the S21 of the normal breathing and the empty measurement data from the VNA. Next, a band pass filter with bandwidth between 0.1 Hz and 3.3 Hz was applied to preserve the vital signs content. Next, the resulting matrix was expressed into a complex form necessary for the CSD method. Further, the FFT was employed to obtain the first and highest peak corresponding to the respiration rate, which occurred at a frequency of 0.3 Hz. The respiration rate is associated with first, second and third harmonics which are usually stronger than the HR. The harmonics were cancelled using a notch adaptive filter with multiple center frequencies at 0.6, 0.9, 1.2 and 1.8 Hz. Lastly, the pulse rate was extracted by searching for the highest peak in the frequency range between 0.7 Hz and 3.3 Hz which includes the human heart rate under resting conditions. In this experiment, the subject were at rest with no external body movement for simplicity purposes.

### 4.3. Results and Discussion

The experimental results are given in Figure 21b,c, for the male and female subjects, respectively. Filtering and background subtraction were used to remove the noise and harmonics of the respiratory signal. A breath rate of 18 breaths per minute and a heart rate of 58 beats per minute were obtained for the male subject. Moreover, these results were 20 breaths per minute and 78 beats per minute, respectively for the female subject.

As seen from the above experiment, CW radars can be used to detect human BR and HR without employing complex signal processing.

## 5. Conclusions

Since human cardio-respiratory rate monitoring is crucial for prediction and diagnosis of cardiovascular and pulmonary diseases, this paper presents methods used for detecting and monitoring these vital signs. The most popular modalities are contact-based with ECG being the gold-standard technique. These methods involve the use of body sensors and cabling, which may not be practical for long-term monitoring. As a result, many recent research activities were focused on contactless-based methods using radar techniques, which do not require the use of electrodes and offer good vital sign detection accuracy. The challenges faced by vital signs radars were discussed along with their state-of-the-art solutions. A proof-of-concept experiment was carried with two different volunteers to demonstrate the potential of Doppler radars in vital signs detection. Even though radars show promising results in detecting human cardio-respiratory rates, the issues of RBM and separation of HR from BR remain the bottleneck of the widespread application of this type of system. The state-of-the-art solutions for motion artifacts usually deal with one-dimensional motions and often exhibit high power consumption. Furthermore, accurate heart rate detection usually requires advanced signal-processing methods. Therefore, future works on vital sign radars are needed for their proliferation in the consumer market. These include more accurate mechanisms for mitigating the 3D motion artifacts of the SUT and less computational loads for vital signs acquisition while keeping the power consumption low.

## Figures and Tables

**Table 1:** Comparison of contact-based vital signs monitoring systems comparing them in terms of vital sign, number of contact needed, its accuracy and major challenges.

	Method		Vital Signs Detected	Minimum Number of Contacts	Accuracy	Long-Term Monitoring	Drawbacks
1	ECG		BR and HR	3	High	Yes	Expensive, MA effect
2	PPG		BR and HR	1	High	Yes	MA, environmental effects
3	Air-based sensing	Air component	BR	1	High	No	Environment effects
		Air temperature	BR	1	High	No	-
		Air humidity	BR	1	High	No	Environmental effects
4	Mechanical displacement sensing of chest	Strain-based	BR	1	High	Yes	Tightly attached probe
		Impedance pneumography	BR	1	High	Yes	MA effect
		3D movement sensing	BR	3	Medium	Yes	Expensive
5	Blood pressure sensing	Non-invasive	HR and BP	1	Medium	Yes	Often requires physician
		Invasive	HR and BP	1	High	No	Clinical uses only
6	PCG		HR	1	High	No	Surrounding sound effects

**Table 2:** Comparison of Radar-Based Vital Signs Monitoring Systems.

Method	Vital Signs Detected	Multi-Subjects Detection	Range Estimation	Power Consumption
CW	BR and HR	No	No	Medium
FMCW	BR and HR	Yes	Yes	High
SFCW	BR and HR	Yes	Yes	Medium
UWB	BR and HR	Yes	Yes	Low

## Abbreviations

The list of abbreviations used in this paper can be found below.

BR	Breath rate
HR	Hearth rate
BP	Blood pressure
OSAS	Obstructive sleep apnea syndrome
SIDS	Sudden infant death syndrome
SUID	Sudden unexpected infant death
WHO	World health organization
COPD	Cognitive obstructive pulmonary disease
HRV	Hearth rate variability
ECG	Electrocardiogram
EDR	ECG-driven respiration
AFE	Analog front-end
DSP	Digital signal processing
MA	Motion artifact
PPG	Photoplethysmogram
LED	Light-emitting-diode
IR	Infrared
DC	Direct-current
VPG	Video-plethysmography
RGB	Red-green-blue
ROI	Region-of-interest
SAW	Surface acoustic wave
FBG	Fiber Bragg grating
AC	Alternative-current
MEMS	Microelectromechanical systems
3D	three-dimensional
PCG	Phonocardiography
CW	Continuous-wave
FPGA	Field-programmable gate array
FMCW	Frequency-modulated Continuous-wave
SFCW	Stepped-frequency continuous-wave
Rx	Receiver
Tx	Transmitter
LO	Local oscillator
IF	intermediate frequency
SSB	Single sideband
DSB	Double sideband
AD	Arctangent Demodulation
CSD	Complex signal demodulation
I	In-phase
Q	Quadrature
RBM	Random body movement
EMD	Empirical mode decomposition
VCO	Voltage-controlled oscillator
PLL	Phase-locked-loop
VNA	Vector network analyzer
UWB	Ultra-wideband
IR-UWB	Impulse radio ultra-wideband
FFT	Fast Fourier transform
IFFT	Inverse Fast-Fourier transform
FCC	Federal communication committee
ECC	European electronic communication commission
SUT	Subject-under-test
ADC	Analog-to-digital converter
VGA	Variable-gain amplifier
